# Polarization-independent isotropic metasurface with high refractive index, low reflectance, and high transmittance in the 0.3-THz band

**DOI:** 10.1515/nanoph-2022-0788

**Published:** 2023-05-31

**Authors:** Kento Sato, Takehito Suzuki

**Affiliations:** Department of Electrical and Electronic Engineering, Graduate School of Engineering, Tokyo University of Agriculture and Technology, #405 Building 5, 2-24-16 Nakacho, Koganei-shi, 184-8588, Tokyo, Japan; Division of Advanced Electrical and Electronics Engineering, Institute of Engineering, Tokyo University of Agriculture and Technology, #405 Building 5, 2-24-16 Naka-cho, Koganei-shi, 184-8588, Tokyo, Japan

**Keywords:** high refractive index, metasurface, polarization-independent property, reflectionless property, terahertz wave

## Abstract

Metasurfaces substituted for naturally occurring materials make it possible to develop flat optics manipulating terahertz waves. However, the control of unprecedented material properties with metasurfaces frequently produces anisotropic material properties and has yet to be commonly adopted because of the limitation of functionalities as optical components. Here, we demonstrate an isotropic metasurface with polarization-independent material properties with the extremely high refractive index of 14.0 + *j*0.49, low reflectance of 1.0 %, and high transmittance of 86.9 % at 0.31 THz. Measurements by terahertz time-domain spectroscopy (THz-TDS) verify that the fabricated metasurface with a high refractive index, low reflectance, and high transmittance works for terahertz waves with any polarization direction and results in the unprecedented material characteristics with polarization independence. The relative permittivity and relative permeability are 13.9 – *j*1.4 and 13.8 + *j*2.3, respectively. The sum of the dielectric and magnetic energy losses must also be considered to verify the conservation of energy for metasurfaces. The sum of the dielectric and magnetic energy losses is very close to positive values and the conservation of energy is largely satisfied. The proposed metasurface would offer optical components with attractive functionalities such as wavefront control, directivity enhancement, and optical vortices for 6G communications.

## 1 Introduction

The manipulation of terahertz waves radiated from terahertz continuous-wave sources is essential for the development of terahertz industrial applications such as 6G wireless communications (Beyond 5G) [1, 2] and imaging [3, 4] The rapid development of accessible terahertz continuous-wave (CW) sources oscillating with a single frequency at room temperature, such as resonant tunnelling diodes (RTD) [5, 6] and quantum cascade lasers (QCL) [7, 8] has covered the commonly called terahertz gap for CW sources. Optical components such as lenses for the control of terahertz waves radiated from terahertz CW sources are frequently made of naturally occurring materials, including cyclo-olefin polymer (COP) with a refractive index 1.5 [9], magnesium oxide (MgO) 3.1 [10], and silicon (Si) 3.4 [11]. The flexible design of material properties is commonly challenging because it is not straightforward to control magnetic properties in naturally occurring materials at high frequencies compared with dielectric properties. Metasurfaces can artificially control permeability as well as permittivity, enabling the flexible design of material properties such as refractive indices, reflectance, and transmittance. The flexible design of material properties with metasurfaces makes it possible to develop unprecedented optical components that can manipulate terahertz waves based on flat optics [12–16].

Our original metasurfaces with a high refractive index of approximately 6.0 and low reflectance with the simultaneous control of both permittivity and permeability [17, 18] have been applied to optical components for the manipulation of terahertz waves with high efficiencies, such as in collimating metalenses [19], focusing lenses [20], and refractive plates [21]. The metasurfaces with a high refractive index and low reflectance are also applied in antennas for 6G wireless communications with directivity enhancement of a single RTD oscillating at 0.3 THz to 4.2 times [22] and integration at the short distance of 1 mm (1 wavelength) from an RTD [23]. However, the previously reported metasurfaces with a high refractive index and low reflectance [17, 18, 24–34] suitable for terahertz oscillators in 6G wireless communications do not achieve isotropic material properties, including the measured relative permittivity and permeability. Anisotropic material properties result in the control of terahertz waves with a specified polarization direction. Isotropic metasurfaces work for terahertz waves with any polarization direction and would further accelerate the development of optical components based on terahertz flat optics. Materials with a high refractive index are helpful for high-resolution imaging, such as solid immersion lenses. The laminated structure of a metasurface with a high refractive index can be an alternative to naturally occurring materials with a high refractive index. Isotropic metasurfaces are essential for imaging because light from objects has random polarizations. Our original previously reported metasurfaces have polarization-dependent anisotropic characteristics [17, 18]. The polarization-independent isotropic metasurface in this manuscript can be applied to polarization-independent metalenses for imaging. Gradient-refractive-index (GRIN) metalenses frequently need meta-atoms with high refractive indices because the distributions of refractive indices increase concentrically from the periphery to the center. The isotropic metasurface in this manuscript would offer an accessible platform for terahertz flat optics in imaging and 6G communications.

In this manuscript, we demonstrate an isotropic metasurface with an extremely high refractive index, low reflectance, and high transmittance in the 0.3-THz band offering artificial materials suitable for planar optical components in 6G wireless communications. The metasurface consists of meta-atoms with square metal patches symmetrically aligned on both the front and back of a dielectric substrate. Measurements by terahertz time-domain spectroscopy (THz-TDS) verify that a metasurface with a high refractive index, low reflectance, and high transmittance works for terahertz waves with any polarization direction and results in material characteristics with polarization independence. The fabricated metasurface has the extremely high refractive index of 14.0 + *j*0.49, reflectance of 1.0 %, and transmittance of 86.9 % for terahertz waves with a specified polarization direction at 0.31 THz. The relative permittivity and relative permeability are 13.9 – *j*1.4 and 13.8 + *j*2.3, respectively. The simultaneous control of both permittivity and permeability with similar values at the same frequency produces an attractive phenomenon with the high transmittance of 86.9 %, while the geometric transmittance is predicted as 34.3 %. The presented isotropic metasurface with an extremely high refractive index, low reflectance, and high transmittance in the 0.3-THz band can directly be applied to polarization-independent planar optical components in the terahertz waveband such as antennas, collimating metalenses, focusing metalenses, refractive plates, and optical vortex phase plates. Terahertz flat optics based on the unprecedented metasurface could be integrated with terahertz oscillators and accelerate the growth of emerging industries such as 6G wireless communications.

## 2 Polarization-independent isotropic metasurface with high refractive index, low reflectance, and high transmittance

**Figure 1:**
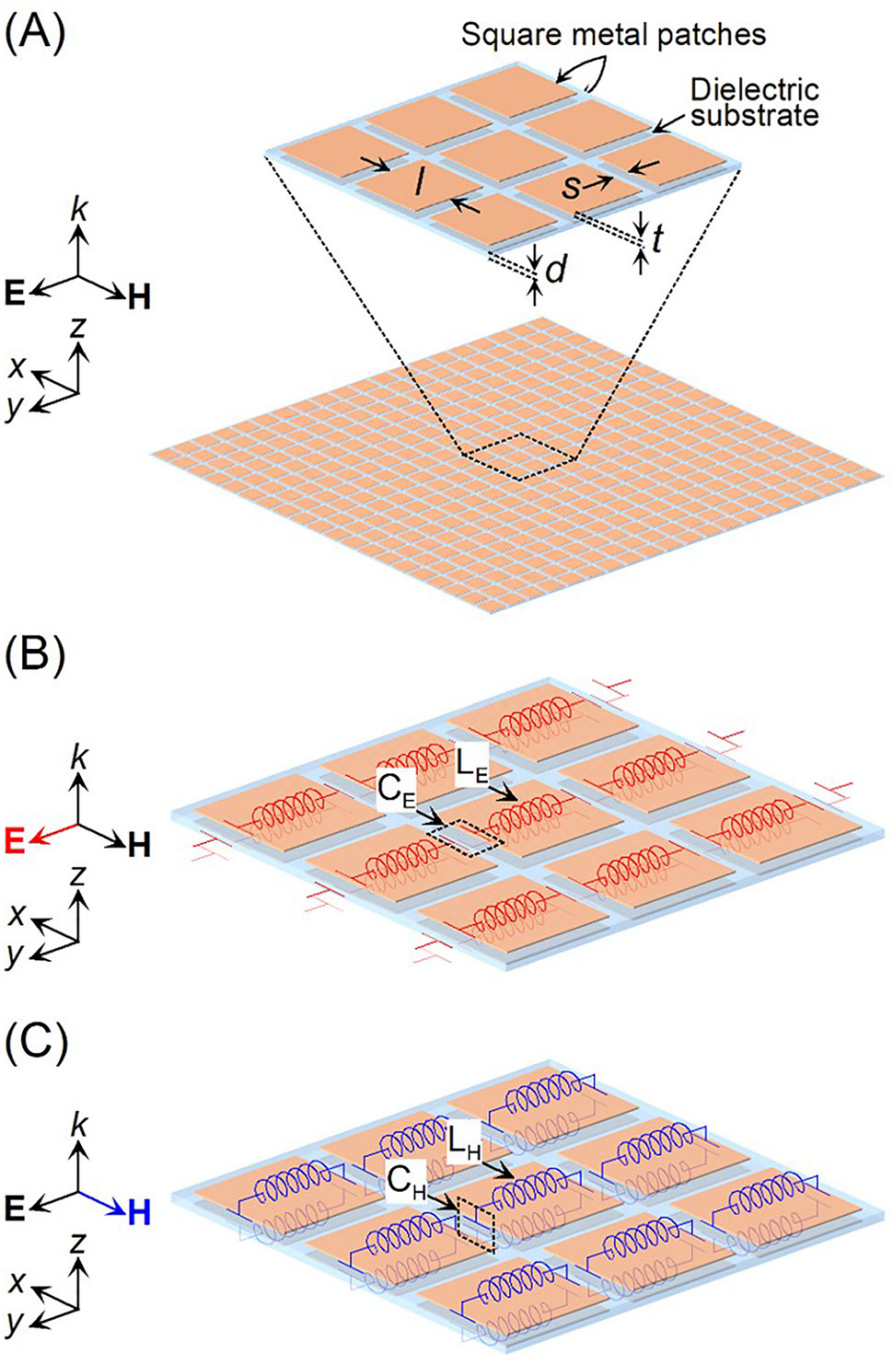
Structure of (A) polarization-independent isotropic metasurface consisting of square metal patches. Equivalent circuits of the polarization-independent isotropic metasurface to explain: (B) dielectric and (C) magnetic properties.

Figure 1(A) illustrates a polarization-independent metasurface with an extremely high refractive index and low reflectance. The metasurface consists of symmetrically aligned meta-atoms with approximately one-third of the wavelength on both the front and back of a dielectric substrate. The meta-atoms of the square metal patches are symmetrical with respect to the *x*-and *y*-axes. The vectors of the electric and magnetic fields forming the incident waves are resolved into two components on the *x*- and *y*-axes. The metasurface consisting of meta-atoms symmetrical with respect to the *x*-and *y*-axes has polarization-independent properties. The operating principle of the polarization-independent metasurface with an extremely high refractive index and low reflectance are described in Figure 1(B) and (C). Figure 1(B) and (C) show the equivalent circuits of a polarization-independent metasurface with an extremely high refractive index and low reflectance to explain the dielectric and magnetic properties, respectively. The electric field of incident terahertz waves generates current on the meta-atoms of the front and back of the dielectric substrate in the same direction. The meta-atom performs as an inductance *L*_E_ and the gaps between the meta-atoms perform as a capacitance *C*_E_, forming an LC series resonant circuit in Figure 1(B). The magnetic field of incident terahertz waves generates current on the meta-atoms of the front and back of the dielectric substrate in the opposite direction. The meta-atom performs as an inductance *L*_H_ and the thickness of the dielectric substrate with the double-sided meta-atoms perform as a capacitance *C*_H_, forming an LC parallel resonant circuit in Figure 1(C). A refractive index $n=\sqrt{\varepsilon_{r}\mu_{r}}$ is high when the relative permeability is close to the relative permittivity with a high value. The relative impedance $Z_{r}=\sqrt{\mu_{r}/\varepsilon_{r}}$ is close to 1.0 resulting in a near zero Fresnel reflection due to the impedance matching from circuit theory when the relative permeability is close to the relative permittivity at the same frequency. The relative permittivity and permeability with a high value are caused by the simultaneous resonance of the dielectric and magnetic properties. The incident terahertz wave in Figure 1 has an electric field along the *y*-axis and a magnetic field along the *x*-axis. The electric and magnetic fields of the incident terahertz wave relate to the dielectric and magnetic properties of the metasurface, respectively. The simultaneous control of dielectric and magnetic resonances at the same frequency can be designed by using the meta-atoms aligned on both the front and back of the dielectric substrate. The dielectric resonance is determined by the inductance components of the meta-atoms and the capacitance components between meta-atoms along the *y*-axis. The magnetic resonance is determined by the inductance components of the meta-atoms and the capacitance component between the front and back of the dielectric substrate.

## 3 Design of polarization-independent isotropic metasurface with high refractive index, low reflectance, and high transmittance

**Figure 2:**
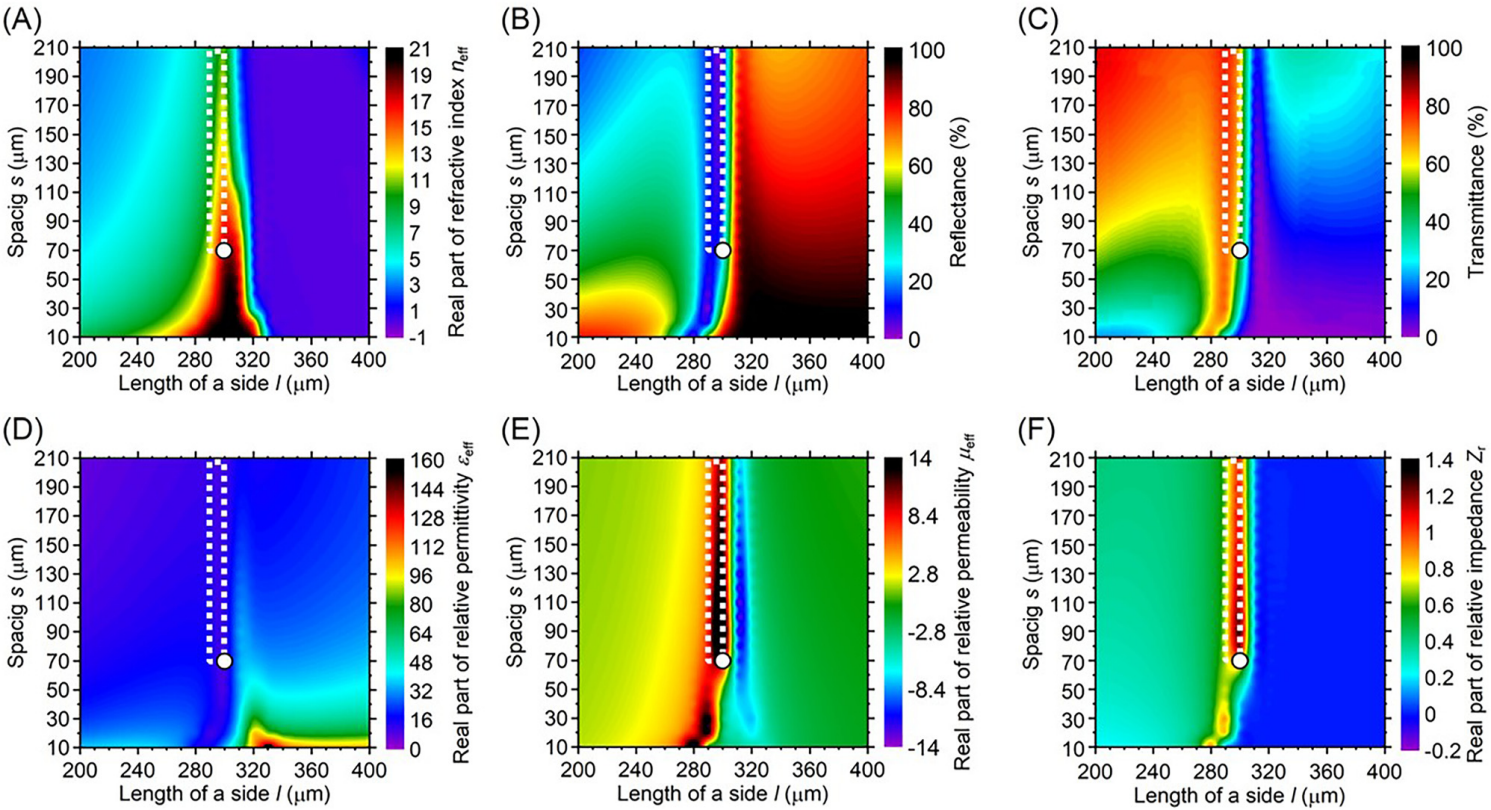
Simulated contour maps for (A) the real parts of refractive indices, (B) reflectance, (C) transmittance, (D) the real parts of relative permittivity, (E) the real parts of relative permeability, and (F) the real parts of relative impedance of the metasurface with square metal patches at 0.31 THz.

Figure 2(A)–(F) show simulated contour maps at 0.31 THz. Figure 2(A) shows the simulated refractive indices, Figure 2(B) the reflectance, Figure 2(C) the transmittance, Figure 2(D) the relative permittivity, Figure 2(E) the relative permeability, and Figure 2(F) the relative impedance. The optimized design of the polarization-independent metasurface with an extremely high refractive index and low reflectance is described in Figure 2. The contour maps show the optical constants varying with square metal patch length *l* from 200 to 400 μm and spacing *s* between the metal patches from 200 to 400 µm. Other parameters are set as the thickness of the dielectric substrate *d* = 23 μm and the thickness of the meta-atom *t* = 0.5 µm. The white dots in Figure 2(A)–(F) show the fabricated parameters of *l* = 300 µm and *s* = 70 µm. The metal of the square patches is copper with a conductivity of 5.8 × 10^7^ S/m. The dielectric substrate is a cyclo-olefin polymer with a measured refractive index of 1.5 + *j*0.001 at 0.5 THz and has low loss properties in the terahertz waveband. The simulated optical constants are derived from scattering matrices *S*_11_ and *S*_21_ of the metasurface obtained from simulations by a finite element method simulator ANSYS HFSS [35]. The simulations in Figure 2(A)–(F) verify that the metasurface consisting of square metal patches of *l* = 290 µm, and *s* = 40 µm has the extremely high refractive index *n*_eff_ of 14.4 + *j*0.70, reflectance of 4.1 %, transmittance of 77.2 %, relative permittivity *ε*_r_ of 18.3 – *j*1.3, relative permeability *μ*_r_ of 11.1 + *j*1.9, and relative impedance *Z*_r_ of 0.78 + *j*0.10.

The metasurface achieves the control of refractive indices with the relative impedance *Z*_r_ = 1, while it is challenging to control the refractive index while maintaining *Z*_r_ = 1. The dimensions of meta-atoms with high refractive indices and *Z*_r_ = 1 are optimized through iterative simulations by the finite element method simulator ANSYS HFSS. The square of the metal path length *l* and spacing *s* are mainly optimized for the design of material properties with high refractive indices and *Z*_r_ = 1. The regions inside the white dotted line in Figure 2(A)–(F) show that the metasurfaces achieve the wide range of refractive indices from 7.1 to 18.0 with a relative impedance *Z*_r_ of approximately 1, resulting in reflectionless material properties. The relative impedance *Z*_r_ changes from 0.62 to 1.4 in the region enclosed by the white dotted line. The design of a relative impedance *Z*_r_ from 0.62 to 1.4 brings about the low 3.5–21.6 % reflectance. The square metal patch length *l* varies from 290 to 300 μm, and the spacing *s* varies from 70 to 210 μm. The low-reflection material properties are caused by the impedance matching, as according to circuit theory both the relative permittivity and permeability have similar values with the optimized design of *l* and *s*. Naturally occurring materials with high refractive indices are frequently subject to high Fresnel reflections because while the relative permittivity can be controlled, the relative permeability cannot commonly be controlled in the terahertz waveband. The metasurfaces in this manuscript make it possible to control both relative permittivity and permeability in the terahertz waveband.

## 4 Measurements and discussion

**Figure 3:**
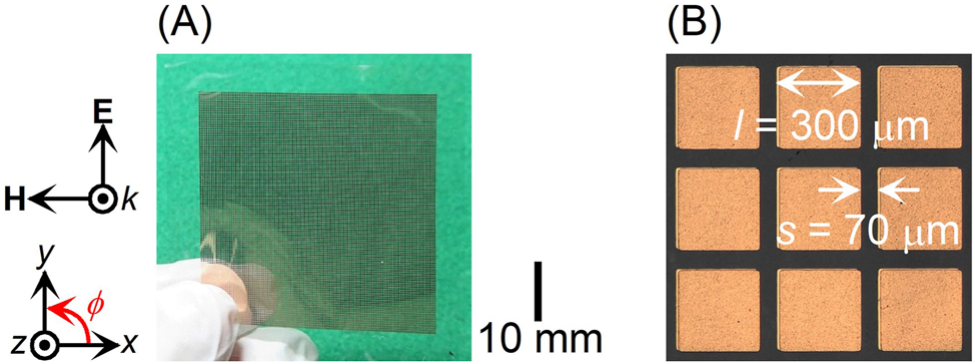
Photograph of (A) fabricated polarization-independent metasurface with an extremely high refractive index and low reflectance at 0.31 THz. (B) Laser microscope image of meta-atoms, square metal patches.

Figure 3(A) is a photograph of the fabricated polarization-independent metasurface with an extremely high refractive index and low reflectance in the 0.3-THz band. The metasurface consists of meta-atoms aligned on both the front and back of a cyclo-olefin polymer film within an area of approximately 40 × 40 mm^2^. The cyclo-olefin polymer film is coated with copper layers on both the front and back. The copper layers are directly coated on the cyclo-olefin polymer film without adhesive layers to reduce conductor losses. The cyclo-olefin polymer film coated with copper layers was etched to fabricate the metasurface. Figure 3(B) shows a laser microscope image of meta-atoms, square metal patches. The metasurface with square metal patches has 11,664 (108 × 108) pairs of double-sided meta-atoms with *l* = 300 µm and *s* = 70 µm in Figure 3(B).

**Figure 4:**
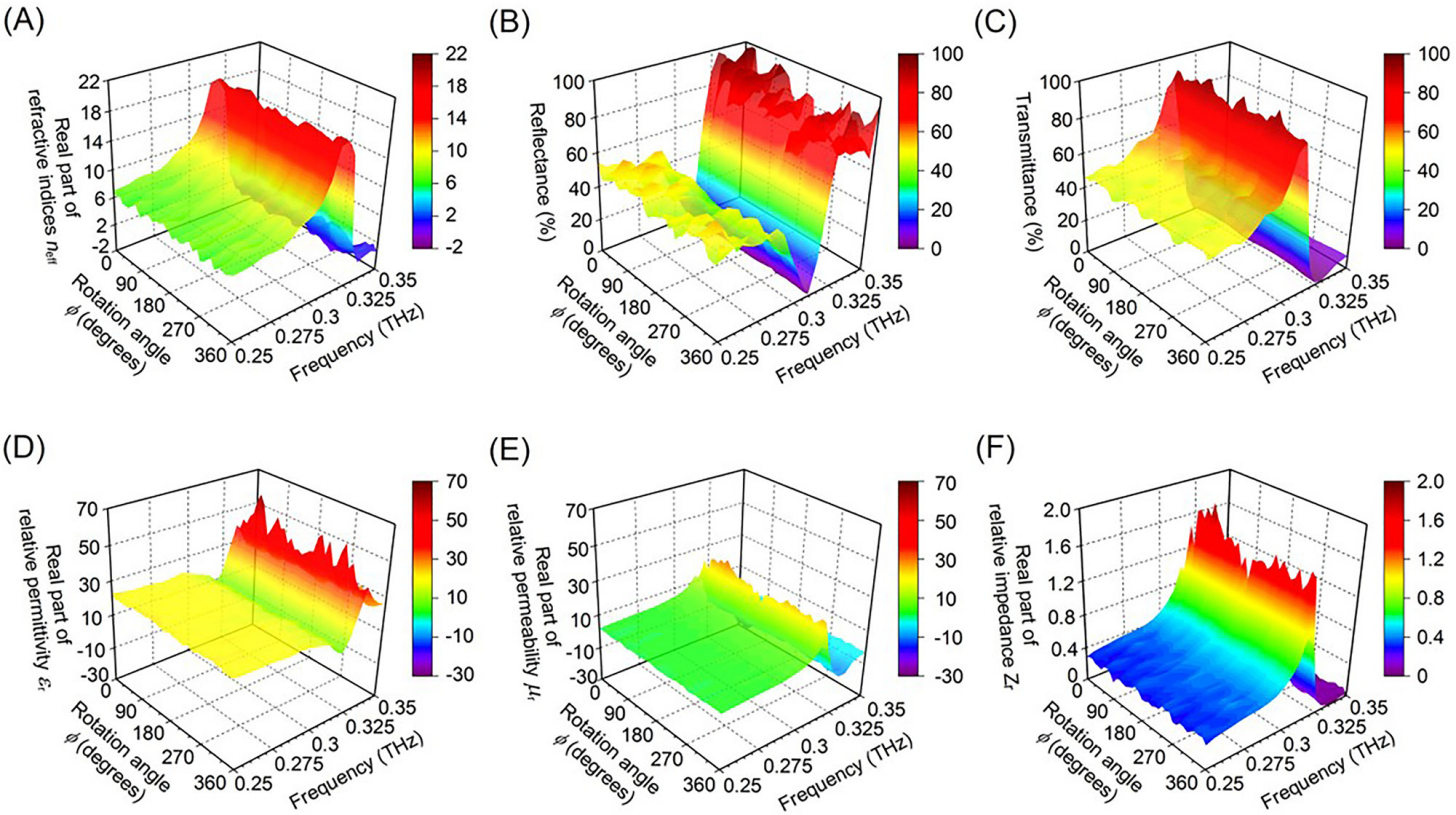
Measured polarization properties for (A) the real parts of refractive indices, (B) reflectance, (C) transmittance (D) the real parts of relative permittivity, (E) the real parts of relative permeability, and (F) the real parts of relative impedance of the fabricated polarization-independent metasurface at 0.31 THz.

Figure 4(A)–(F) show measured contour maps at 0.31 THz. Figure 4(A) shows the measured refractive indices, Figure 4(B) the reflectance, Figure 4(C) the transmittance, Figure 4(D) the relative permittivity, Figure 4(E) the relative permeability, and Figure 4(F) the relative impedance. The measured optical constants are derived from scattering matrices *S*_11_ and *S*_21_ of the metasurface obtained from measurements by THz-TDS TOPTICA TeraFlash [35]. The scattering matrices *S*_11_ (reflection components) and *S*_21_ (transmission components) are calculated from the transmitted and reflected time waveforms of the fabricated metasurface and references using discrete Fourier transforms. The reference conditions in the transmission and reflection measurements are dry air and a silver mirror, respectively. The optical length of the transmission measurements for the fabricated metasurface is the same as that of the reference dry air. Differences in the optical lengths of the fabricated metasurface and silver mirror in the reflection measurements are unavoidable because of deflection of the fabricated metasurface. The reflection measurements are compensated for on the condition that the phases of the reflection components S_11_ measured by THz-TDS are the same as the phases simulated by the ANSYS HFSS. The comparison between the measurements by THz-TDS and simulations by ANSYS HFSS by the compensation predicts that the fabricated metasurface with square metal patches is concave with a depth of 255 µm along the transmitted terahertz waves. The polarization properties are measured by rotating the metasurface with a rotation angle on the *xy*-plane from 0° to 360° in 10° steps. Figure 4(A)–(F) verifies that the metasurface with square metal patches has polarization-independent properties in the 0.3-THz band. Measurements demonstrate that the metasurface with square metal patches has the extremely high refractive index of 14.0 + *j*0.49, low reflectance of 1.0 %, and high transmittance of 86.9 % at 0.31 THz for a rotation angle *ϕ* = 0° in Figure 4(A)–(C). The measured relative permittivity and relative permeability are 13.9 – *j*1.4 and 13.8 + *j*2.3, respectively, at 0.31 THz in Figure 4(D) and (E). The similar high real parts of the relative permittivity and permeability cause the impedance matching based on circuit theory, resulting in a relative impedance of 0.99 + *j*0.13 close to 1.0 at 0.31 THz in Figure 4(F). The area ratio of the gaps between the meta-atoms to the spot size of terahertz waves, the geometric transmittance, is 34.3 %. The area ratio of the meta-atoms to the spot size of the terahertz waves is 65.7 %. The measurements also demonstrate that the simultaneous control of relative permittivity and permeability with similar high values causes the high transmittance of 86.9 % while the geometric transmittance is predicted at 34.3 %. The bandwidth of the polarization-independent isotropic metasurface with a refractive index above Si (3.4) is 0.18 THz, and the fractional bandwidth is 58.1 %. Further, our work predicts that the total frequency characteristics of metalens antennas were saturated and broader than those of metasurfaces because metalens antennas consisted of polarization-dependent anisotropic meta-atoms with a wide range of refractive indices from high to low values in a previous paper [22]. A metalens antenna made with the polarization-independent isotropic metasurface presented here would have a bandwidth to satisfy the specifications in 6G wireless communications. A gradient-refractive-index collimating metalens antenna consisting of the square metal patches would enhance the directivity of terahertz CW sources [36]. The polarization-independent metasurface in the 0.3-THz band can be applied to that in the 50-THz band [37]. The fabricated metasurface in the 50-THz band consists of 1200 nm × 1200 nm square Au patches periodically arranged with a 200-nm spacing on both the front and back of a 100-nm thick SiN_x_ membrane. The polarization-independent metasurface may be applied to the control of thermal radiation with unpolarized terahertz emissions.

**Figure 5:**
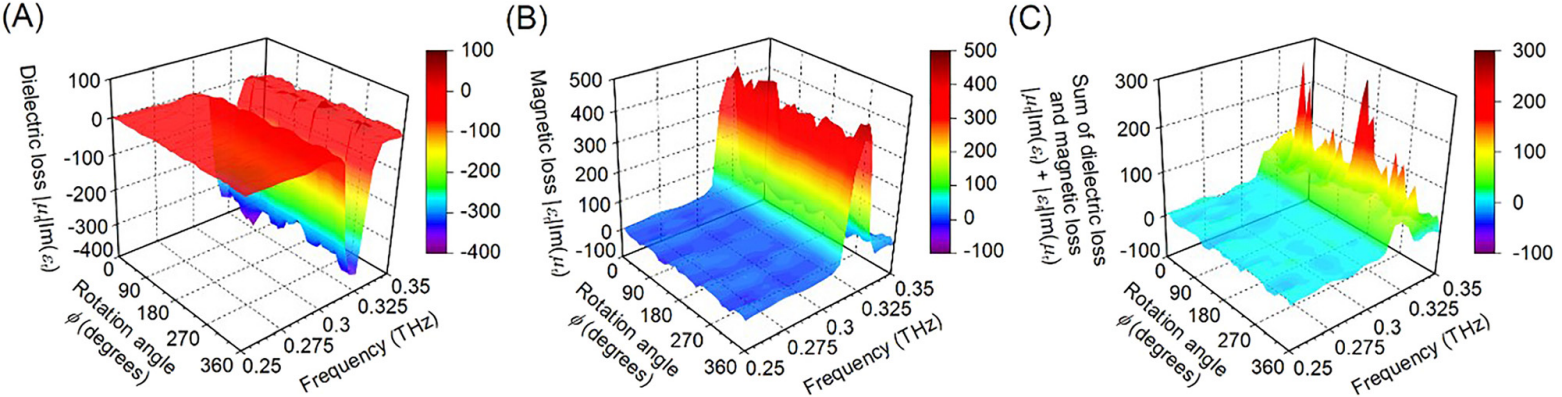
Measured (A) dielectric energy loss and (B) magnetic energy loss of the fabricated polarization-independent metasurface. (C) Measured summantion of dielectric energy loss and magnetic energy loss of the fabricated polarization-independent metasurface.

Figure 5(A) and (B) show measured dielectric energy |*μ*_r_|Im(*ε*_r_) and magnetic energy losses |*ε*_r_|Im(*μ*_r_), respectively. Figure 5(C) shows measured sum of the dielectric energy and magnetic energy losses |*μ*_r_|Im(*ε*_r_) + |*ε*_r_|Im(*μ*_r_) [38] of the fabricated polarization-independent metasurface. Figure 5(A) shows that the measured dielectric energy loss has negative values. Figure 5(B) shows that the measured magnetic energy loss has positive values. Figure 5(C) shows that the sum of the dielectric and magnetic energy losses is very close to positive values and the conservation of energy is largely satisfied.

## 5 Summary

In this manuscript, we experimentally demonstrate a polarization-independent reflectionless metasurface with an extremely high refractive index, low reflectance, and high transmittance in the 0.3-THz band. The metasurface consists of symmetrically aligned square metal patches on both the front and back of a dielectric substrate. Measurements by THz-TDS verify that a fabricated isotropic metasurface has polarization-independent material properties with the extremely high refractive index of 14.0 + *j*0.49, reflectance of 1.0 %, and transmittance of 86.9 % at 0.31 THz. The resonance of the dielectric and magnetic properties produces material properties with the high relative permittivity of 13.9 – *j*1.4 and the high relative permeability of 13.8 + *j*2.3. The simultaneous resonance of the dielectric and magnetic properties causes a measured transmittance of 86.9 % which is much higher than the geometric transmittance predicted as 34.3 %. The presented polarization-independent metasurface will offer an accessible material platform for terahertz flat optics. Planar optical components in flat optics based on the unprecedented metasurface would offer attractive functionalities such as wavefront control, directivity enhancement, and optical vortices and be a welcome contribution to the growing industries such as 6G wireless communications.
